# Identification of polyunsaturated fatty acids as potential biomarkers of osteoarthritis after sodium hyaluronate and mesenchymal stem cell treatment through metabolomics

**DOI:** 10.3389/fphar.2023.1224239

**Published:** 2023-08-15

**Authors:** Qinyan Yang, Yiran Zhao, Na Li, Jian-Lin Wu, Xiaolun Huang, Mei Zhang, Xiqing Bian, Yi-Zhun Zhu

**Affiliations:** ^1^ School of Pharmacy, Macau University of Science and Technology, Taipa, Macao SAR, China; ^2^ State Key Laboratory for Quality Research of Chinese Medicine, Macau University of Science and Technology, Taipa, Macao SAR, China; ^3^ Liver Transplant Center and HBP Surgery, Sichuan Cancer Hospital and Institute, Sichuan Cancer Center, School of Medicine, University of Electronic Science and Technology of China, Chengdu, China

**Keywords:** osteoarthritis, sodium hyaluronate, mesenchymal stem cells, metabolomics, derivatization–liquid chromatography–mass spectrometry, correlation analysis

## Abstract

**Introduction:** Osteoarthritis (OA) is a prevalent joint disorder worldwide. Sodium hyaluronate (SH) and mesenchymal stem cells (MSCs) are promising therapeutic strategies for OA. Previous studies showed they could improve knee function and clinical symptoms of OA. However, the mechanism of the therapeutic effects on the improvement of OA has not been clearly explained.

**Methods:** In our study, we used a technique called 5-(diisopropylamino)amylamine derivatization liquid chromatography coupled with mass spectrometry to find the metabolites in OA synovial fluid under different treatments.

**Results and Discussion:** After looking into the metabolomics, we discovered that SH and MSC treatment led to the downregulation of ω-6 polyunsaturated fatty acids (PUFAs) and the upregulation of ω-3 PUFAs. Significantly, the contents of 5(S)-HETE, PGA2, PGB2, and PGJ2 were lower in the MSC group than in the SH group after quantification using 5-(diisopropylamino)amylamine derivatization–UHPLC–QQQ-MS. This is the first report on the relationship of 11(S)-HETE, PGA2, PGB2, PGF2β, 11β-PGF2α, and DK-PGE2 with OA. Moreover, the correlation analysis of metabolites and inflammation factors showed the positive association of ω-6 PUFAs with pro-inflammation cytokines, and of ω-3 PUFAs with anti-inflammation cytokines. Our results indicated the therapeutic effect of SH and MSCs in patients with OA. In addition, this reliable metabolic approach could uncover novel biomarkers to treat OA.

## 1 Introduction

Osteoarthritis (OA) is one of the most prevalent joint disorders worldwide (Arra et al., 2022). OA patients often have inflammation and limited joint movements, which can impact their daily activities (Mustonen and Nieminen, 2021). OA is regarded as a severe disease that significantly affects society and brings a heavy burden on humans. OA can be treated with lubricant supplements or surgeries, such as microfracture and drilling. However, they could only ameliorate the symptoms. Moreover, the drilling method is invasive (Chiang et al., 2016). Treating OA with intra-articular administrations of sodium hyaluronate (SH) or mesenchymal stem cells (MSCs) was promising. They could improve knee function, reduce cartilage loss, and slow down the progression of OA (Lu et al., 2022). However, the improvement of OA only relies on the reduction of signs, and the mechanism has not been clearly explained. Thus, it is necessary to hunt for novel targets for the elucidation of the therapeutic mechanism and, finally, to combat the influence of OA on human life.

Here, metabolomics was applied to elucidate the mechanism. Carboxylic acids were reported to be related to OA (Loef et al., 2019). They play essential roles in the response to inflammation, which have important contributions to the pathogenic mechanism of OA (Loef et al., 2019). It was reported that saturated fatty acids could cause inflammation by inducing macrophages to secrete tumor necrosis factor α (TNF-α) (Van de Vyver et al., 2020). Unsaturated fatty acids were also related to OA and could increase the gene expression linked to cartilage degradation (Loef et al., 2020; MacFarlane et al., 2020). Therefore, the profiling of carboxylic acids is essential for the analysis of the influence of SH and MSCs on the treatment of OA.

Scientists used different methods to find carboxylic acids in different biological samples, such as gas chromatography–mass spectrometry (GC–MS) and liquid chromatography–mass spectrometry (LC–MS) (Zarate et al., 2017; Gu et al., 2018; Giusepponi et al., 2019). However, the high reaction temperature and the instability of the acids limited GC–MS application. Additionally, it is difficult to directly analyze these metabolites through LC–MS due to the low endogenous content in biological samples and the poor ionization efficiency. Derivatization was applied to solve the aforementioned difficulties (Bian et al., 2017; Bian et al., 2020; Xiang et al., 2020). For example, 5-(dimethylamino)naphthalene-1-sulfonyl piperazine (Xiang et al., 2020) and 2,4-bis(diethylamino)-6-hydrazino-1,3,5-triazine (Hu et al., 2017) were used to derivatize carboxylic acids. However, the sensitivities were not high enough to analyze the trace-level acids, e.g., prostaglandins. Recently, in our previous study (Bian et al., 2018; Bian et al., 2020), a method called 5-(diisopropylamino)amylamine (DIAAA) derivatization–LC–MS was used to detect carboxylic acids in serum with high sensitivity.

Thus, we used this technique to profile carboxylic acids in the synovial fluid from healthy and OA guinea pigs treated with MSCs or SH, or nothing. The potential biomarkers could be determined through various statistical analyses. Then, DIAAA derivatization–UHPLC–QQQ-MS was applied to quantify these biomarkers. Moreover, the relationships of metabolites with inflammation factors were also elucidated. The results would help to explain the mechanism of the therapeutic effect of the drugs on OA and help diagnose and provide a prognosis for OA.

## 2 Materials and methods

### 2.1 Chemicals and reagents

We purchased all polyunsaturated fatty acid (PUFA) standards from Cayman Chemical (Ann Arbor, MI). The chemicals (1-[bis(dimethylamino)methylene]-1*H*-1,2,3-triazolo[4,5-b]pyridinium 3-oxid hexafluorophosphate (HATU), DIAAA, 1-hydroxybenzotriazole hydrate (HOBt), and triethylamine (TEA)) were bought from Sigma-Aldrich Laboratories Inc. (St. Louis, MO). Acetonitrile (HPLC grade) was obtained from Anaqua Chemicals Supply Inc., Ltd. (Houston, TA). A Millipore water purification system (Billerica, MA) was applied to prepare deionized water. Sigma-Aldrich Laboratories Inc. provided other chemical reagents and formic acid (MS grade).

### 2.2 Preparation of solutions

The stock solutions of the PUFA mixture were obtained by dissolving the corresponding standards in 60% ACN (MS grade) at 1 μg/mL concentration. In addition, 12(S)-HETE, 5(S)-HETE, and 12(S)-HHTrE were made at different concentrations to create a wider range of linearity. Different concentrations of working solutions (Supplementary Table S1) were used to create the calibration curves. HOBt (20 mM) and HATU (20 mM) were dissolved in DMSO, while DIAAA-TEA solution was dissolved in DMSO to reach the final concentration of 100 mM. All solutions were stored at −20°C before use.

### 2.3 Culture and isolation of adipose mesenchymal stem cells

The adipose tissue was initially washed with 0.9% normal saline (NS). Then, the solution of type I collagenase (0.75 mg/mL) was added, and it was digested for 30 min in a thermostatic shaker at 37°C. It was isolated after centrifugation for 10 min at 900 g, and the remaining pellet, which is the stromal vascular fraction (SVF), was retrieved. The SVF was washed with 0.9% NS and mixed well. After centrifugation (300 g, 10 min), the supernatant was abandoned, the residue was re-suspended in 0.9% NS, and the suspension of the cell was filtered with a cell filter (100 μm). Then, the cell was counted and distributed evenly into two centrifuge tubes. The next step was the primary inoculation of MSCs. In brief, they were inoculated in 2 × 10^6^/T75 vials, replenishing the medium to reach the volume of 15 mL/vial. The incubation conditions of the cells were 37°C with 5% CO_2_. The medium was changed after 48 h of incubation and then changed every 3 days. When the cells’ confluence reached 80–90%, they were absorbed and passaged with 0.125% pancreatic enzyme. Injection therapy was performed using P5 generation cells.

### 2.4 Animal modeling and articular cavity injection

The protocol of the experiment was authorized by the Macau University of Science and Technology ethical committee. Guinea pigs (Hartley, weighing 350–400 g, 2 months old) were recruited for the study. After being fed to get adapted to the environment for 1 week, these guinea pigs were randomly divided into four groups, which included the control (N = 4), model (N = 8), SH (N = 7), and MSC (N = 7) groups. For the model, SH, and MSC groups, partial meniscus resection was performed on the left knee joint. After modeling, the guinea pigs were fed for 2 weeks, and injection into the knee cavity was performed by percutaneous puncture. Group A (N = 8) was injected with normal saline (100 μL) into the joint cavity. Group B (N = 7) was injected with 100 μL adipose mesenchymal stem cell suspension (1 × 10^7^, generation P5). Group C (N = 7) was injected with 100 μL SH. Then, synovial fluid samples were collected after 3 weeks. Then, the hair around the left knee joint of guinea pigs was scraped and the skin was disinfected with medical alcohol. The guinea pigs were fixed on the operating table with adhesive tape, and 100 μL of the synovial fluid was extracted from their knee by percutaneous puncture with a microsyringe (Hamilton). The samples were kept at −80°C before use.

### 2.5 Enzyme-linked immunosorbent assay test

The synovial fluid samples from each group were thawed at room temperature. ELISA was used to check the contents of TNF-α, transforming growth factor β (TGF-β), TNF-γ, interleukin 1β (IL-1β), IL-1, IL-4, matrix metalloproteinase 1 (MMP1), MMP13, ADAMTS-4, and ADAMTS-5 in the synovial fluid of guinea pigs.

### 2.6 Sample preparation

In all, 50 µL synovial fluid samples were extracted with 200 µL cold methanol, and then, they were centrifuged at 13,500 rpm for 5 min at 4°C. The residues were extracted twice more, and then, the supernatants were combined and dried with a nitrogen stream. The residues were kept at −20°C before DIAAA derivatization.

### 2.7 Derivatization

The derivatization was accomplished according to our previously published literature (Bian et al., 2018; Bian et al., 2020). In brief, 5 μL of 20 mM HOBt, 5 μL of 100 mM DIAAA-TEA solution, and 5 μL of 20 mM HATU were added to the dried residue and left for 1 min at room temperature. Then, 35 μL ACN was added to make it 50 μL in total.

### 2.8 UHPLC–Q-TOF/MS analysis

An Agilent 1,290 Infinity LC system (UHPLC, Santa Clara) was employed for this study. A Waters ACQUITY UPLC HSS T3 column (2.1 × 100 mm, 1.8 μm) was applied to separate the metabolites. The temperature of the column was kept at 40°C, and the autosampler was maintained at 4°C. The flow rate was set as 0.3 mL min^−1^. Mobile phase A was 0.1% formic acid-containing water, and mobile phase B was 0.1% formic acid-containing acetonitrile. The gradient was 0–0.5 min, 2%–5% B; 0.5–2.5 min, 5%–6% B; 2.5–3.5 min, 6%–7% B; 3.5–4.3 min, 7%–7.3% B; 4.3–7.3 min, 7.3%–7.8% B; 7.3–10 min, 7.8%–9% B; 10–12 min, 9%–14% B; 12–17 min, 14%–23% B; 17–18 min, 23%–26% B; 18–20 min, 26%–47% B; 20–23 min, 47%–85%; 23–24 min, 85%–94% B; 24–27.9 min, 95% B; and 28 min, 2% B.

An Agilent 6550 UHD accurate-mass Q-TOF/MS system was used for mass integration. The positive (POS) ion mode was applied for analysis. These were the MS parameters used: the dry gas flow at 15 L min^−1^, the dry gas temperature at 250°C, sheath gas flow at 11 L min^−1^, sheath gas temperature at 300°C, nebulizer pressure at 20 psig, nozzle voltage at 500 V, and capillary voltage at 5000 V. The mass spectra range was *m/z* 100–1,000. They used a low-frequency TOF reference mixture [containing the internal reference masses at *m/z* 922.0098 (C_18_H_18_F_24_N_3_O_6_P_3_)] for accurate mass measurements. Automated and targeted MS/MS were elucidated for MS/MS acquisition, and the collision cell energy (CE) was set at 30 eV.

### 2.9 UHPLC–QQQ-MS analysis

The metabolites were separated by using an Agilent 1,290 Infinity LC system (UHPLC, Santa Clara, CA), which consisted of a thermostatted column compartment, an autosampler, and a binary pump. The column was Waters ACQUITY UPLC HSS T3 (2.1 × 100 mm, 1.8 μm). The autosampler was set at 4°C. For the quantification of PUFAs, the gradient was established with the modification to shorten the analytical time (Supplementary Table S2).

MassHunter Quantitative Analysis B.06.00 (Agilent Technologies, Santa Clara, CA) was applied to analyze the results. The MRM mode was recruited to obtain the MS/MS data. The parameters of mass were dry gas temp 250°C, dry gas flow 13 L/min; nebulizer 25 psi, sheath gas temp 275°C, sheath gas flow 11 L/min, capillary 3500 V, and nozzle voltage 500 V. The software Optimizer (Agilent Technologies, Santa Clara, United States) was applied to optimize the specific transitions of MRM and the corresponding CE of the targeted PUFAs. The optimal parameters of MRM for each metabolite are shown in Table 1.

**TABLE 1:** Linearity and LOQs of PUFAs using DIAAA derivatization–UHPLC–QQQ-MS.

No.	Metabolites	Precursor ions	Product ions	CE (eV)	LOQ (ng/mL)	Linear range (ng/mL)	R^2^
1	15(S)-HETE	489.4	471.4	24	0.0135	0.0135–30	0.997
2	11(S)-HETE	489.4	471.4	24	0.0041	0.0041–30	0.998
3	12(S)-HETE	489.4	471.4	24	0.0014	0.0014–30	0.995
3^*^	12(S)-HETE	489.4	471.4	24	-	125–10,000	0.992
4	5(S)-HETE	489.4	471.4	24	0.0041	0.0041–30	0.995
4^*^	5(S)-HETE	489.4	471.4	24	-	10–400	0.997
5	15(S)-HEPE	487.4	469.5	24	0.0135	0.0135–30	0.992
6	12(S)-HEPE	487.4	469.5	24	0.0135	0.0135–30	0.995
7	5(S)-HEPE	487.4	469.5	24	0.041	0.041–30	0.995
8	PGA2	503.4	443.3	34	0.0216	0.0216–48	0.996
9	PGB2	503.4	443.3	34	0.0216	0.0216–32.4	0.995
10	PGJ2	503.4	443.3	34	0.0216	0.0216–48	0.998
11	PGD2	521.4	503.4	30	0.027	0.027–40.5	0.997
12	PGE2	521.4	503.4	30	0.014	0.014–72.9	0.998
13	PGE1	523.4	505.5	30	0.027	0.027–60	0.997
14	PGF2α	523.4	505.5	30	0.027	0.027–60	0.998
15	PGF2β	523.4	505.5	30	0.027	0.027–40.5	0.994
16	6-keto-PGF1α	539.4	521.4	24	0.054	0.054–270	0.992
17	11β-PGF2α	523.4	505.5	30	0.027	0.027–60	0.996
18	13,14-Dihydro-15-keto PGE2	521.4	86.2	50	0.027	0.027–60	0.998
19	15(S)-HETrE	491.5	473.3	24	0.0007	0.0007–30	0.997
20	12(S)-HHTrE	449.4	86.1	46	0.54	0.54–400	0.998
20^*^	12(S)-HHTrE	449.4	86.1	46	-	50–2,000	0.991
21	13(S)-HOTrE	463.4	445.5	56	0.0135	0.0135–30	0.993
22	TXB2	539.4	521.4	24	0.18	0.18–270	0.996

*Calibration curve constructed for high concentration.

### 2.10 Data analysis

MassHunter software (Agilent Technologies) was used to extract and align raw data for analysis. The metabolites were confirmed by comparing their MS/MS spectra with the standards from METLIN (https://metlin.scripps.edu/index.php) and LIPID MAPS (http://www.lipidmaps.org/), or by analyzing the MS/MS spectra. Orthogonal projections to latent structures discriminant analysis (OPLS-DA) and biplot were recruited to evaluate the correlation of metabolites among different groups by using SIMCA-P software (version 14.0; Umetrics, Umea, Sweden). The volcano plot was processed using Instant Clue (Nolte et al., 2018). TBtools (https://github.com/CJ-Chen/TBtools/releases) was used to perform the heatmap. MetaboAnalyst 5.0 was applied to construct pathway enrichment analysis. MassHunter Quantitative Analysis software was used to integrate the areas of peaks, which were measured through UHPLC/QQQ-MS. The discrimination between two different groups was calculated by using GraphPad Prism 9.0. The difference with *p* < 0.05 was significant (*, *p* < 0.05; **, *p* < 0.01; and ***, *p* < 0.001). Fold change was calculated by comparison of the SH group or MSC group with the OA guinea pigs (model) group, and the healthy (control) group with the model group. Fold change with a positive value shows a relatively higher concentration in the SH group, MSC group, or control group, while a negative value indicates a relatively lower concentration compared to the control group.

## 3 Results

### 3.1 Assessment of the therapeutic effect of SH and MSC through the International Cartilage Repair Society and magnetic resonance imaging

**FIGURE 1:**
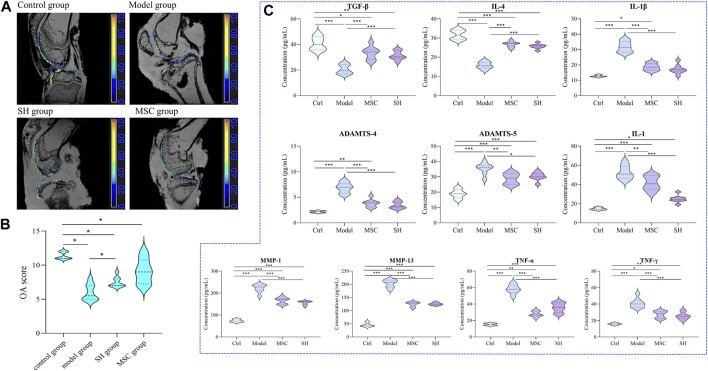
Assessment of the therapeutic effect of MSC and SH on OA through MRI **(A)** and ICRS **(B)**; dynamic changes of inflammation factors among different groups **(C)** (*, *p* < 0.05; **, *p* < 0.01; and ***, *p* < 0.001).

It is known that the International Cartilage Repair Society (ICRS) classification is important to assess lesion stability. Magnetic resonance imaging (MRI) is widely used to appraise OA lesions. The appearance of guinea pigs’ cartilage plateau was evaluated using ICRS and MRI (Figures 1A,B). For the control group, the surface of the normal articular cartilage is clear and smooth, with complete morphology and uniform thickness. For the model group, it suffered serious cartilage injury and the joint cavity was deformed. The SH group treatment increased articular cartilage intensity but caused slight joint inflammation. However, after treatment with MSC (MSC group), the appearance was similar to that in the SH group. Especially, no obvious inflammation could be seen in the MSC group.

### 3.2 Changes in inflammation factors among the different groups

Considering that the development of OA involved immune cells, which are the hot spot issue in recent years, the contents of inflammation factors, such as tumor necrosis factor α (TNF-α), interleukin 1β (IL-1β), transforming growth factor β (TGF-β), TNF-γ, IL-1, IL-4, matrix metalloproteinase 1 (MMP1), MMP13, ADAMTS-4, and ADAMTS-5, were detected by ELISA in the synovial fluid of each group. The results showed that these inflammation factors had significant differences comparing the model group with that in the control, MSC, and SH groups (Figure 1C; Supplementary Figure S1). Especially, the contents of TNF-α, IL-1β, IL-1, MMP1, MMP13, ADAMTS-4, and ADAMTS-5 showed significant decreases in the control group, as well as in the MSC or SH groups. This indicated that the expression of these inflammatory factors was significantly inhibited after the treatment by MSC or SH. However, the levels of IL-4 and TGF-β significantly upregulated in the MSC or SH groups. The results manifested that the expression of the two factors was significantly activated after the treatment by MSC or SH.

### 3.3 Metabolomics study of different groups

#### 3.3.1 Metabolomics study of SH, control, and model groups

**FIGURE 2:**
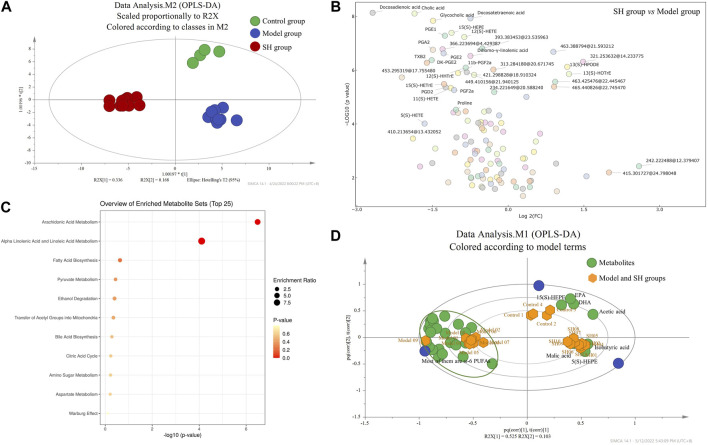
OPLS-DA **(A)** and biplot **(D)** among control, model, and SH groups; volcano plot **(B)** and pathway enrichment analysis **(C)** between the model and SH groups (2D: green dots represent the metabolites, and yellow dots represent the samples including control, model, and SH groups).

Then, metabolomics was applied to try to elucidate the mechanism of SH in the treatment of OA in our study. The synovial fluid was collected and applied for the metabolomics study as it could directly reflect the development of OA. The synovial fluid was initially extracted and dried under the nitrogen stream, and then, the residues were derivatized and analyzed using UHPLC-Q-TOF/MS. Molecular features were extracted using MassHunter qualitative analysis software (Agilent Technologies) and explained with SIMCA-P software (version 14.0; Umetrics, Umea, Sweden). We found significantly different metabolic profiles after comparing the control group levels with model and SH group levels using OPLS-DA (Figure 2A). Furthermore, the differential metabolites, which were identified using OPLS-DA, were performed for volcano plot analysis between the SH and model groups (Figure 2B); 116 metabolites showed significant differences (|Fold change| > 1.5; *p* < 0.05), and the levels of 87 metabolites decreased in the SH groups, whereas others increased compared to those in the model group. Furthermore, the differential metabolites were analyzed between model and control groups, and 42 metabolites that changed in the same way in the control and SH groups were found (Supplementary Table S3), suggesting they might give some indicators to treat OA using SH. After comparing the MS with that of standards, the structures of 33 metabolites were confirmed, and they were mainly related to various metabolic pathways like arachidonic acid metabolism, α-linolenic and linoleic acid metabolism, fatty acid biosynthesis, bile acid biosynthesis, and pyruvate metabolism (Figure 2C).

To further examine the overall trends, a biplot was used to identify which metabolites contributed the most to the variance in the control, model, and SH groups. The correlation plot (Figure 2D) manifested that eicosapentaenoic acid (EPA), docosahexaenoic acid (DHA), and 5(S)- and 15(S)-HEPEs, which are ω-3 PUFAs, were related to the control and model groups, while the ω-6 PUFAs, particularly 5(S)-, 11(S)-, 12(S)-, and 15(S)- HETEs, 11β-PGF2α, 6-keto-PGF1α, DK-PGE2, DK-PGD2, PGA2, PGB2, PGE2, PGD2, PGF2α, PGF2β, and PGE1, were related to the model group. In addition, these metabolites were significantly reduced in the SH and control groups compared to the model group (Supplementary Table S3).

#### 3.3.2 Metabolomics study of the MSC, control, and model groups

**FIGURE 3:**
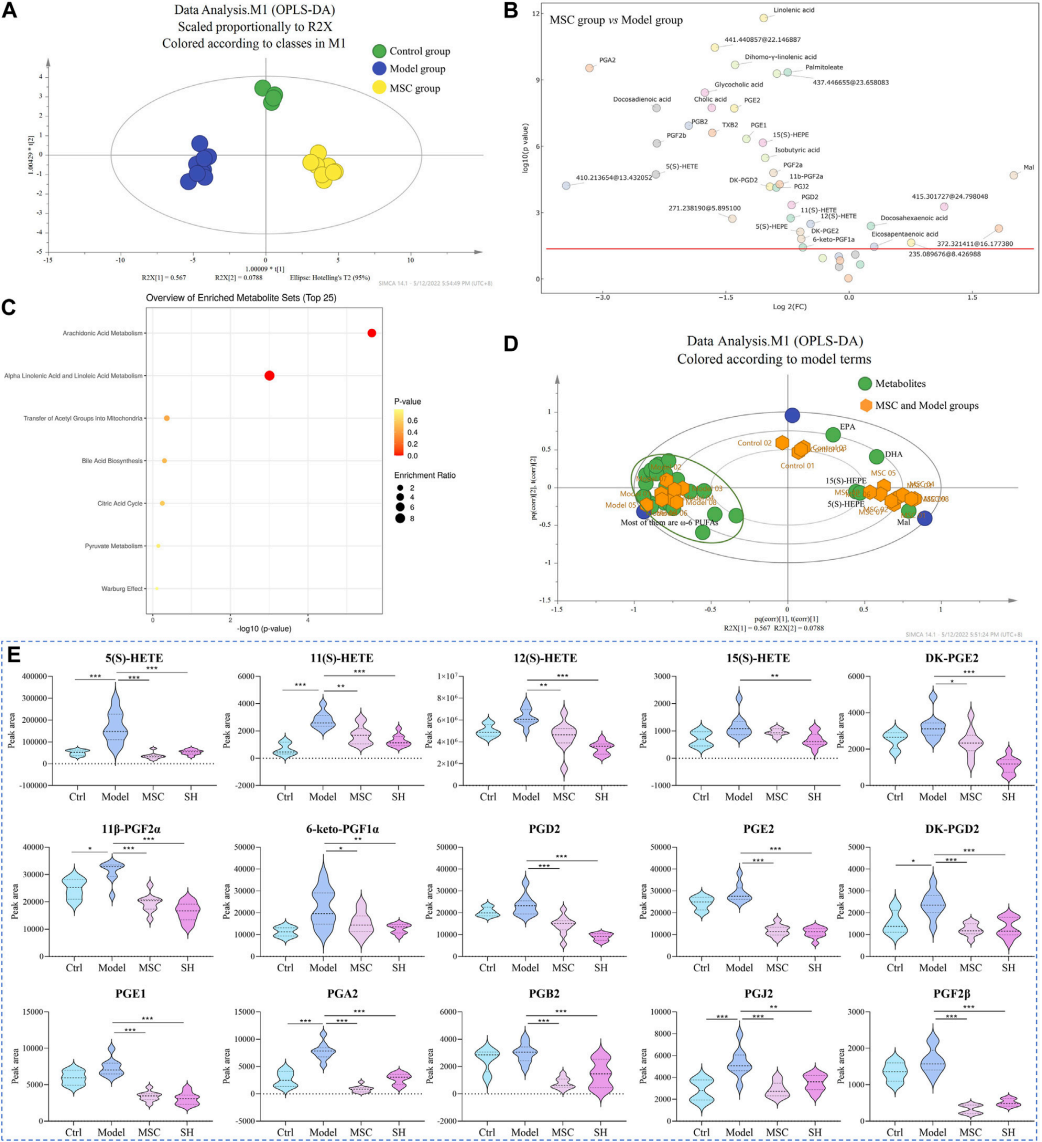
OPLS-DA **(A)** and biplot **(D)** among control, model, and MSC groups; volcano plot **(B)** and pathway enrichment analysis **(C)** between the model and MSC groups (3D: green dots represent the metabolites, and yellow dots represent the samples including control, model, and MSC groups); **(E)** dynamic changes of representative metabolites among different groups (*, *p* < 0.05; **, *p* < 0.01; and ***, *p* < 0.001).

As the metabolites mentioned previously showed significant differences in the SH group, we examined their changes among the MSC, control, and model groups and whether their changes could explain the mechanism of MSC treatment on OA. Here, the abovementioned 42 metabolites were extracted and analyzed to elucidate the influence of MSC treatment on OA. Through OPLS-DA, we found that MSC, model, and control groups could separate dramatically from each other (Figure 3A). Furthermore, the analysis of the volcano plot (Figure 3B) showed that 36 metabolites have significant differences between the model and MSC groups (*p* < 0.05). A total of 34 metabolites (Supplementary Table S4) had similar changing trends in the MSC/model group compared with the control/model group and 27 of their structures were confirmed (Supplementary Table S4). The biplot (Figure 3D) also manifested that several ω-6 PUFAs, e.g., 5(S)-, 11(S)-, 12(S)-, and 15(S)-HETE, 11β-PGF2α, PGF2β, PGA2, PGB2, PGE2, and PGE1, were closely related to the model group. Additionally, these metabolites had lower expression levels after MSC treatment compared to the model group (Supplementary Table S4), whereas ω-3 PUFAs were associated with MSC and control groups (Figure 3D).

The results indicated that after treatment with MSC or SH, these metabolites (representative metabolites are presented in Figure 3E, and others are shown in Supplementary Figure S1), which were associated with arachidonic acid metabolism, could provide some indications that MSC or SH had therapeutic effects on OA. It was reported that PUFAs and their metabolites are beneficial and essential factors for the health of bone (Abshirini et al., 2021). They play important roles in the structure of the cell membrane and act as lipid-mediated signaling molecules’ precursors, which may impact bone remodeling (Abshirini et al., 2021; Mustonen et al., 2021). Thus, the determination and quantification of PUFAs are crucial for the mechanism study of OA. In our following studies, DIAAA derivatization (Bian et al., 2018; Bian et al., 2020) coupled with the UHPLC–QQQ-MS method would be applied to sensitively and efficiently quantify PUFAs in the synovial fluid from OA guinea pigs, which were treated with MSC or SH or nothing.

### 3.4 Quantification of PUFAs in the control, model, MSC, and SH groups

#### 3.4.1 Optimization of the parameters and conditions of UHPLC–QQQ-MS

We optimized and confirmed MRM transition parameters for each derivatized PUFA using their respective commercial standards through online software. The suitable MRM ion channels were established and the corresponding CEs were optimized to find the best values. Each compound was determined with one quantifier ion, as is displayed in Table 1.

#### 3.4.2 Method validation

##### 3.4.2.1 Linearity and limits of quantification

The linearity was assessed by the correlation coefficient at 12 concentrations. All analytes obtained good linearity with R^2^ values higher than 0.99. The limits of quantification (LOQs) of the analytes were between 0.0007 and 0.54 ng/mL. The sensitivity was significantly increased up to 149-fold using DIAAA derivatization–UHPLC–QQQ-MS analysis compared to the literature (Pedersen et al., 2021). As the contents of PUFAs, especially prostaglandins, were lower in biological samples (Quehenberger et al., 2010), DIAAA derivatization–UHPLC–QQQ-MS was a suitable approach to quantify them in the synovial fluid sensitively.

##### 3.4.2.2 Accuracy and precision

The precision and accuracy were analyzed with high, middle, and low concentrations (C3, C5, and C7 in Supplementary Table S1). Accuracy was calculated via the percentage of a measured value to the actual value, and the precision was determined as the relative standard deviation (RSD) of injections three times in a single day or three consecutive days. We found that the accuracy rate was between 80% and 114% and that of precision was less than 20% (Supplementary Table S5). The results manifested that the conventional method could precisely and accurately quantify PUFAs.

##### 3.4.2.3 Matrix effect and recovery

Matrix effect and recovery in serum had been reported in our previously reported literature (Bian et al., 2018). We evaluated them in the synovial fluid in this study. The matrix effects were between 80.14% and 118.37% (Supplementary Table S6), indicating that we did not find significant matrix effects in the matrix. The recovery was between 80.07% and 119.16% (Supplementary Table S6), indicating that PUFA quantification was not affected by other components in the matrix.

#### 3.4.3 Quantification of PUFAs in the synovial fluid

**FIGURE 4:**
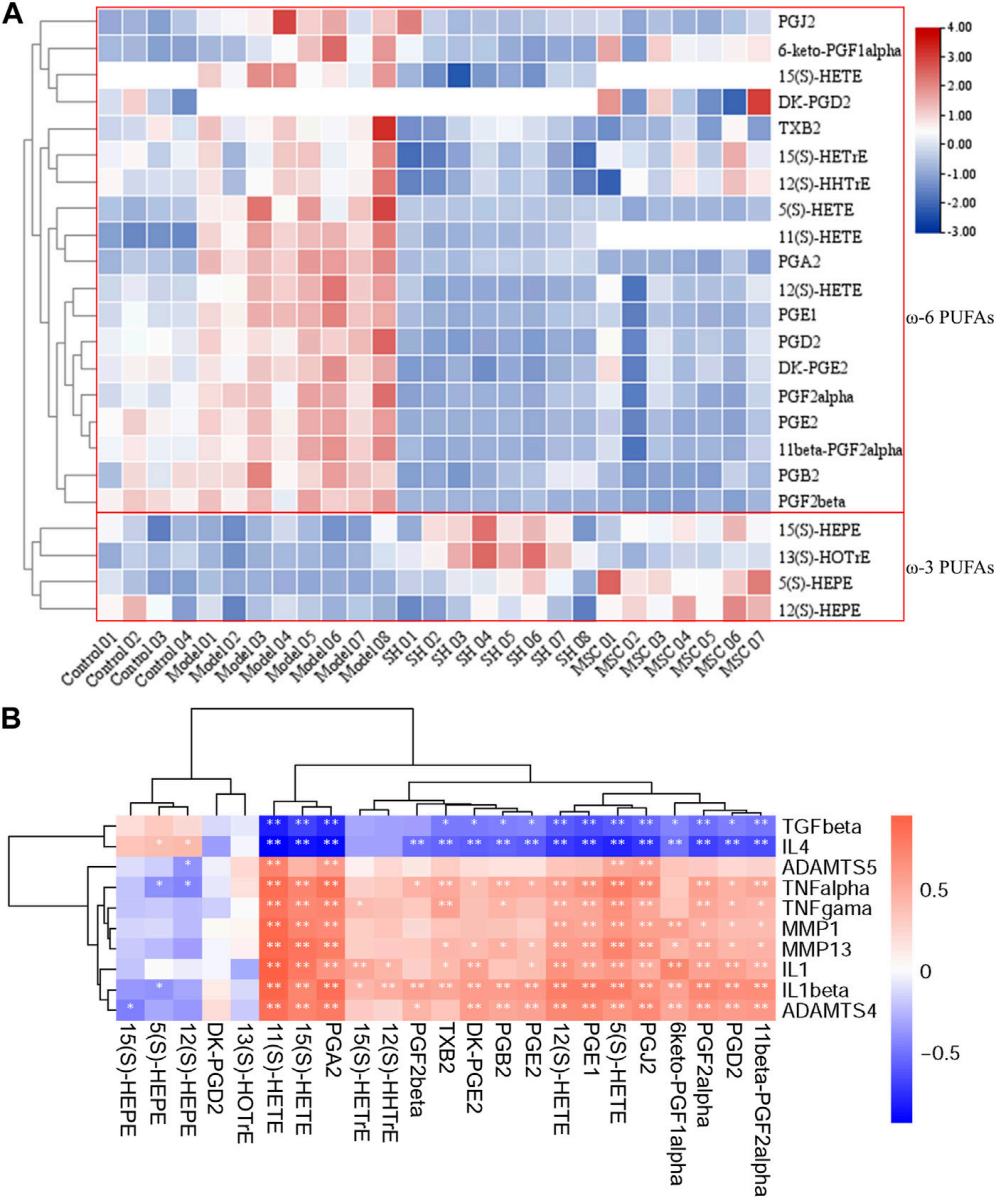
Heatmap of PUFAs after DIAAA derivatization–UHPLC–QQQ-MS analysis **(A)**; relationship of PUFAs with inflammation factors **(B)** (*, *p* < 0.05 and **, *p* < 0.01).

PUFAs in the synovial fluid from control and OA guinea pigs, which were treated with SH or MSC or neither, respectively, were quantified using the developed approach. Their concentrations, except 5(S)-HETE, 12(S)-HETE, and 12(S)-HHTrE, ranged from 0.034 to 24.49 ng/mL (Table 2). In addition, the contents of 5(S)-HETE, 12(S)-HETE, and 12(S)-HHTrE were relatively higher and ranged from 35.19 to 8,897.64 ng/mL (Table 2). The heatmap in Figure 4A shows a distinguished clustering among the ω-6 and ω-3 PUFAs. The contents of ω-6 PUFAs were higher in the model group compared with those in the control, SH, and MSC groups. Conversely, the levels of ω-3 PUFAs, including 5(S)-, 12(S), and 15(S)-HEPEs and 13(S)-HOTrE, were lower in the model group. The statistical analysis (Supplementary Table S7) indicated that ω-6 PUFAs, which were associated with arachidonic acid metabolism (Supplementary Figure S2), had significant differences in the model group compared with other groups. For example, the content of 5(S)-HETE was 219.65 ng/mL in the model group. After SH and MSC treatment, its contents decreased to 56.84 and 35.19 ng/mL, respectively. However, the levels of PGA2 decreased about 3.74- and 10.60-fold after SH and MSC treatment compared with those in the model group. Significantly, the contents of 15(S)- and 11(S)-HETEs were 1.52 and 3.84 ng/mL in the model group, respectively. After MSC treatment, they could not be detected (Table 2).

**TABLE 2:** Concentration of PUFAs (ng/mL) in the synovial fluid of the control, model, SH, and MSC groups.

Name	Control group		Model group		SH group		MSC group	
	Concentration	RSD (%)	Concentration	RSD (%)	Concentration	RSD (%)	Concentration	RSD (%)
15(S)-HETE	-	-	1.52	17.82	0.76	31.66	-	-
11(S)-HETE	0.44	24.96	3.84	16.53	1.42	21.38	-	-
12(S)-HETE	5,572.46	7.03	8,897.64	16.23	3,773.31	12.66	4,803.63	17.72
5(S)-HETE	44.19	18.52	219.65	26.69	56.84	6.99	35.19	26.16
PGA2	0.10	20.13	0.53	15.72	0.14	25.99	0.050	25.57
PGB2	0.14	23.63	0.21	18.93	0.062	24.64	0.042	29.23
PGJ2	0.12	10.47	0.38	14.70	0.22	27.59	0.15	24.62
PGD2	0.084	6.06	0.11	18.32	0.039	9.85	0.060	22.10
PGE2	0.12	13.46	0.14	14.27	0.047	7.42	0.046	15.29
PGE1	0.58	13.14	1.02	14.08	0.33	16.88	0.32	19.46
PGF2α	2.85	10.71	4.43	18.99	1.61	18.36	1.85	17.56
PGF2β	0.17	11.90	0.19	20.39	0.050	10.46	0.034	27.09
6-keto-PGF1α	0.23	15.31	0.65	29.62	0.29	23.37	0.61	23.79
11β-PGF2α	0.17	9.60	0.22	16.19	0.089	9.79	0.093	24.62
DK-PGE2	0.33	15.58	0.44	16.72	0.12	26.92	0.21	20.34
15(S)-HETrE	6.87	8.16	7.68	16.67	4.97	21.41	7.02	14.91
12(S)-HHTrE	1,251.48	9.07	1,484.54	19.27	891.53	23.48	1,264.95	16.11
TXB2	19.65	11.57	24.49	21.67	16.53	21.08	15.41	24.01
5(S)-HEPE	0.46	8.28	0.41	8.81	0.70	27.97	1.04	23.57
12(S)-HEPE	9.47	24.07	7.51	13.98	7.66	23.59	11.09	12.25
15(S)-HEPE	0.21	21.26	0.19	15.90	0.27	22.63	0.26	10.82
13(S)-HOTrE	6.17	14.03	5.54	19.47	11.56	22.39	6.96	11.76

### 3.5 Associations between PUFAs and inflammation factors in OA

To further study the influence of the metabolism on the changes of inflammatory patterns in OA, the relationships of the abovementioned metabolites with inflammation factors in OA models, as well as in the control, SH, and MSC groups, were analyzed (Figure 4B).

A previous report showed that IL-1β and MMP13 could increase prostaglandin production in the synovium after the damage to the articular cartilage (Sanchez-Lopez et al., 2022). Interestingly, our results also showed positive relationships of prostaglandins with IL-1β and MMP13. In addition, consistent with the decreased levels of ω-6 PUFAs after MSC or SH treatment, the inflammatory markers (TNF-α, TNF-γ, IL-1β, IL-1, MMP1, MMP13, ADAMTS-4, and ADAMTS-5) displayed positive correlations with ω-6 PUFAs, such as 5(S)-, 8(S)-, 11(S)-, 12(S)-, and 15(S)-HETEs, PGA_2_, PGB_2_, PGJ_2_, PGE_2_, and PGD_2_, and a negative association with ω-3 PUFAs. However, TGF-β and IL-4 were inversely associated with ω-6 PUFAs and positively related to ω-3 PUFAs, e.g., 5(S)-, 12(S)-, and 15(S)-HEPEs.

## 4 Discussion

OA is a degenerative cartilage disease, and inflammation is believed to be an important factor in the development of OA (Robinson et al., 2016). It was reported that some pro-inflammatory mediators, e.g., TNF-α, IL-1β, IL-6, and IL-15, increased in the synovial fluid or serum of OA patients compared to healthy controls (Philp et al., 2017). PUFAs and their metabolites are bio-active lipid mediators, which mainly include prostaglandins and lipoxins, and are generated from arachidonic acid through lipoxygenase (LOX) and cyclooxygenase (COX) pathways. They can regulate inflammation (Korotkova and Jakobsson, 2014) and affect bone remodeling (Abshirini et al., 2021; Mustonen et al., 2021).

SH injection is a traditional treatment that can lessen pain, improve joint function, and enhance rheological properties (Nishida et al., 2021). In addition, stem cell therapy, which uses MSCs, promises to prevent the degeneration of the OA process (Zhang et al., 2022). Studies have shown that MSCs can reduce inflammation, improve joint function, and slow down cartilage degeneration (Hong et al., 2019; Lopa et al., 2019). However, the mechanism of their treatments on OA has not been fully studied, and in particular, their effects on the progression of OA through metabolites are not yet known.

A carboxylic acid metabolomics study was performed on the synovial fluid samples from different groups using DIAAA derivatization–UHPLC–Q-TOF/MS. Metabolomics and multivariate analyses revealed that arachidonic acid metabolism showed significant differences in the SH or MSC groups compared with that in the model group. Especially, the levels of ω-6 PUFAs, including 5(S)-, 11(S)-, 12(S)-, and 15(S)-HETEs, PGA2, PGB2, PGD2, PGE2, PGE1, DK-PGE2, PGF2α, 11β-PGF2α, and PGJ2, decreased in the synovial fluid after SH or MSC treatment. However, the trends of ω-3 PUFAs, especially 5(S)-, 12(S)-, and 15(S)-HEPEs, EPA, and DHA, increased after SH or MSC treatment in comparison with those in the model group. The levels of prostaglandins are higher in those with rheumatoid arthritis (RA) or OA (Abdolmaleki et al., 2020; Mustonen and Nieminen, 2021). Additionally, the report stated that HETE contents, which are metabolized via LOX enzymes from arachidonic acid, increased in the synovial fluid from RA patients (Wang et al., 2021). Conversely, ω-3 PUFAs, DHA and EPA, can reduce inflammation by changing lipid mediator profiles or producing cytokines and T-cell reactivity (Calder, 2017; Veselinovic et al., 2017). Our results also found higher levels of EPA and DHA in the SH or MSC groups compared with the model group.

In order to further study the therapeutic effect of SH or MSC on OA, the targeted PUFAs were quantified using the method called DIAAA derivatization–UHPLC–QQQ-MS. Finally, we found that the contents of ω-6 PUFAs, including 5(S)-, 11(S)-, 12(S)-, and 15(S)-HETEs, PGA2, PGB2, PGD2, PGE2, PGE1, DK-PGE2, PGF2α, 11β-PGF2α, and PGJ2, decreased almost 1.7–3.8-folds and they had significant differences between the model and SH groups, as well as between the model and MSC groups. In addition, the contents of 5(S)-HETE, PGA2, PGB2, and PGJ2 were lower in the MSC group compared with those in the SH group. Especially, 11(S)-HETE and 15(S)-HETE could not be detected with MSC treatment. It was well known that 15-HETE, which was metabolized from arachidonic acid through 15-LOX, showed high expression under the actions of 15-LOX in chondrocytes (Attur et al., 2015; Tian et al., 2021). 12(S)-HETE was also reported to be related to several inflammatory and degenerative diseases, and its level was found to be increased in OA (Kelly et al., 2015). Prostaglandins, including PGE2, PGA2, PGB2, and PGD2, are metabolized from arachidonic acid via COX-2 (Yui et al., 2015). It was reported that PGE2 promotes OA progression by being secreted through the subchondral bone tissue of OA patients’ osteoblast lineage (Jiang et al., 2022). It was shown that PGD2, PGF2α, and 6-keto-PGF1α were increased in joints with OA (Kojima et al., 2004; Guan et al., 2018; Valdes et al., 2018). In addition, other studies have indicated that MSCs are able to modulate the differentiation of mature dendritic cells and macrophages toward reparative and anti-inflammatory profiles and downregulated pro-inflammatory expression. COX-2 and PGE2 play key roles in MSC-mediated myeloid modulation (Ortiz-Virumbrales et al., 2020). These details were associated with our results that these metabolites were higher in the model group than in the control group. Thus, SH and MSC may help treat OA since they have lower levels of ω-6 PUFAs.

As we know, OA synovial fluid contains different kinds of metabolites and inflammatory factors that affect cartilage injury and inflammation (Anderson et al., 2018). Statistical analysis showed strong connections between the metabolites and inflammatory factors. In brief, it was clearly found that ω-6 PUFAs displayed a positive correlation with TNF-α, TNF-γ, IL-1β, IL-1, MMP1, MMP13, ADAMTS-4, and ADAMTS-5 and were negatively related to TGF-β and IL-4. Differently, ω-3 PUFAs showed converse relationships with these factors. In fact, inflammation is featured by the increased contents of pro-inflammatory cytokines, e.g., IL-1, 6, 8, 1β, TNF-α, and they could influence the activity of osteoclast and thus affect the resorption of bone. The presence of ADAMTS-4 and -5 was also reported to be related to cartilage injuries. MMPs are involved in the digestion of cartilage collagen and are highly expressed in OA (Mehana et al., 2019). Our results also found increased levels of MMP-1 and -13 in the model group compared with the control, MSC, and SH groups. IL-1β led to increased levels of cyclooxygenase-2 (COX-2) (Wojdasiewicz et al., 2014), which finally caused the increased trends of prostaglandins in the model group. IL-1β and MMP13 could stimulate PG production (Crofford et al., 1994). IL-4, which is a pivotal cytokine in cartilage, has been reported to have lower expression in the model group (Assirelli et al., 2014). Given that IL-4 could hamper the release of MMP-13 (Assirelli et al., 2014), the lower IL-4 in OA could cause higher levels of MMP-13, which is in accordance with our results. TGF-β is necessary for cartilage integrity and could avoid damage to the cartilage (Thielen et al., 2021). After treatment with MSC or SH, the contents of TGF-β increased. Considering that ω-6 PUFAs cause inflammation and ω-3 PUFAs reduce it, the relationship of PUFAs with inflammation factors clearly illustrated the mechanism of OA, which is treated with MSC or SH.

Although our manuscript is helpful in confirming the therapeutic effect of SH or MSC in patients with OA, there are also several limitations to our study. First, because of the lack of available clinical data, we could not further validate our results. Second, we only studied guinea pigs aged 3–4 months and created the OA injury model ourselves, without considering other factors such as natural aging. Additional studies are needed to test other conditions. In addition, a proper normalization strategy could be considered in our future work. Due to the low volume of the synovial fluid and the instability of ALOX15, COX-1, and COX-2, it is hard to detect them. We will enrich the volume and detect them immediately when we obtain the samples in future. The other potential limitation is that we investigated only male guinea pigs to reduce other influences (e.g., hormone and menstrual cycle). In future, we may study female guinea pigs as well. Finally, we should expand the sample size to further confirm our results.

## 5 Conclusion

In our study, PUFAs had significant differences among different groups after metabolomics analysis. Especially, patients who received SH or MSC treatment showed decreased levels of pro-inflammatory ω-6 PUFAs and increased levels of anti-inflammatory ω-3 PUFAs. In addition, 11(S)-HETE, PGA2, PGB2, PGF2β, 11β-PGF2α, and DK-PGE2 were identified as OA-related for the first time. Our results showed that the mechanism of SH and MSC treatment could be elucidated through the differential changing levels of PUFAs. In other words, this reliable metabolic approach could uncover novel metabolites, which could help confirm the therapeutic effect of SH or MSC in patients with OA.

## Data Availability

The datasets presented in this study can be found in online repositories. The names of the repository/repositories and accession number(s) can be found at: https://www.ebi.ac.uk/metabolights/, MTBLS6169.
