# Supplementary material for: Identification of polyunsaturated fatty acids as potential biomarkers of osteoarthritis after sodium hyaluronate and mesenchymal stem cell treatment through metabolomics
Source: Front Pharmacol. 2023 Aug 15;14:1224239. doi: 10.3389/fphar.2023.1224239 (PMC10462907; doi:10.3389/fphar.2023.1224239)
Supplement: Supplementary file 1 [file Table1.DOCX]

**Identification of poly-unsaturated fatty acids as potential biomarkers of osteoarthritis after sodium hyaluronate and mesenchymal stem cells treatment through metabolomics**

Qinyan Yang^1,2,3^, Yiran Zhao^2^, Na Li^2^, Jian-Lin Wu^2^, Xiaolun Huang^1,2,3^, Mei Zhang^1,2^, Xiqing Bian^1,2,*^, Yi-Zhun Zhu^1,2,*^

^1^School of Pharmacy, Macau University of Science and Technology, Taipa, Macao.

^2^State Key Laboratory for Quality Research of Chinese Medicine, Macau University of Science and Technology, Taipa, Macao.

^3^Liver Transplant Center and HBP Surgery, Sichuan Cancer Hospital & Institute, Sichuan Cancer Center, School of Medicine, University of Electronic Science and Technology of China, Chengdu, China.

Corresponding author: Xiqing Bian, Ph.D.; Yi-Zhun Zhu, Professor

Email: xiqingbian@163.com (X. Bian); yzzhu@must.edu.mo (Y.Z. Zhu)


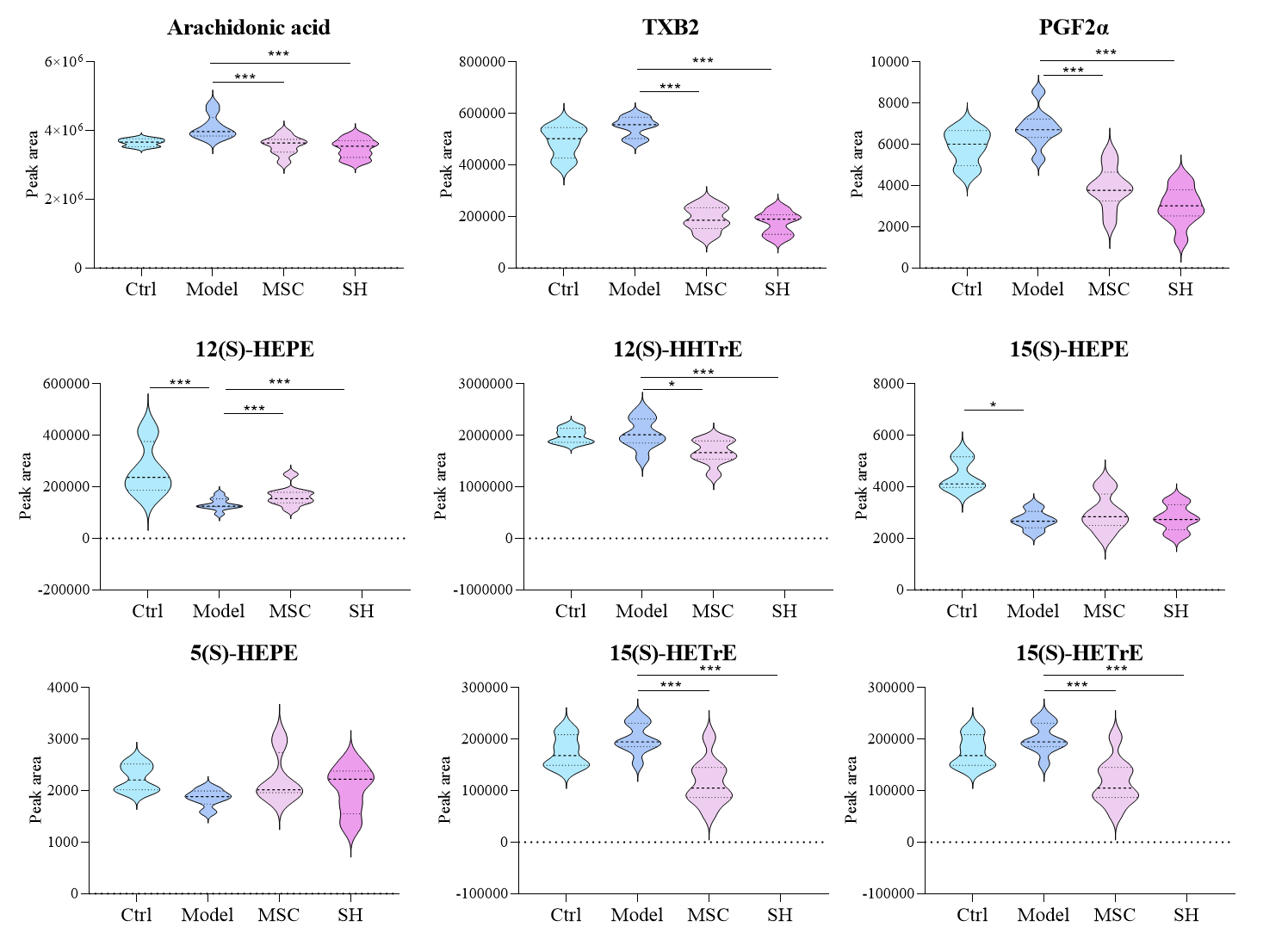


**Figure S1**. The changes of PUFAs among different groups


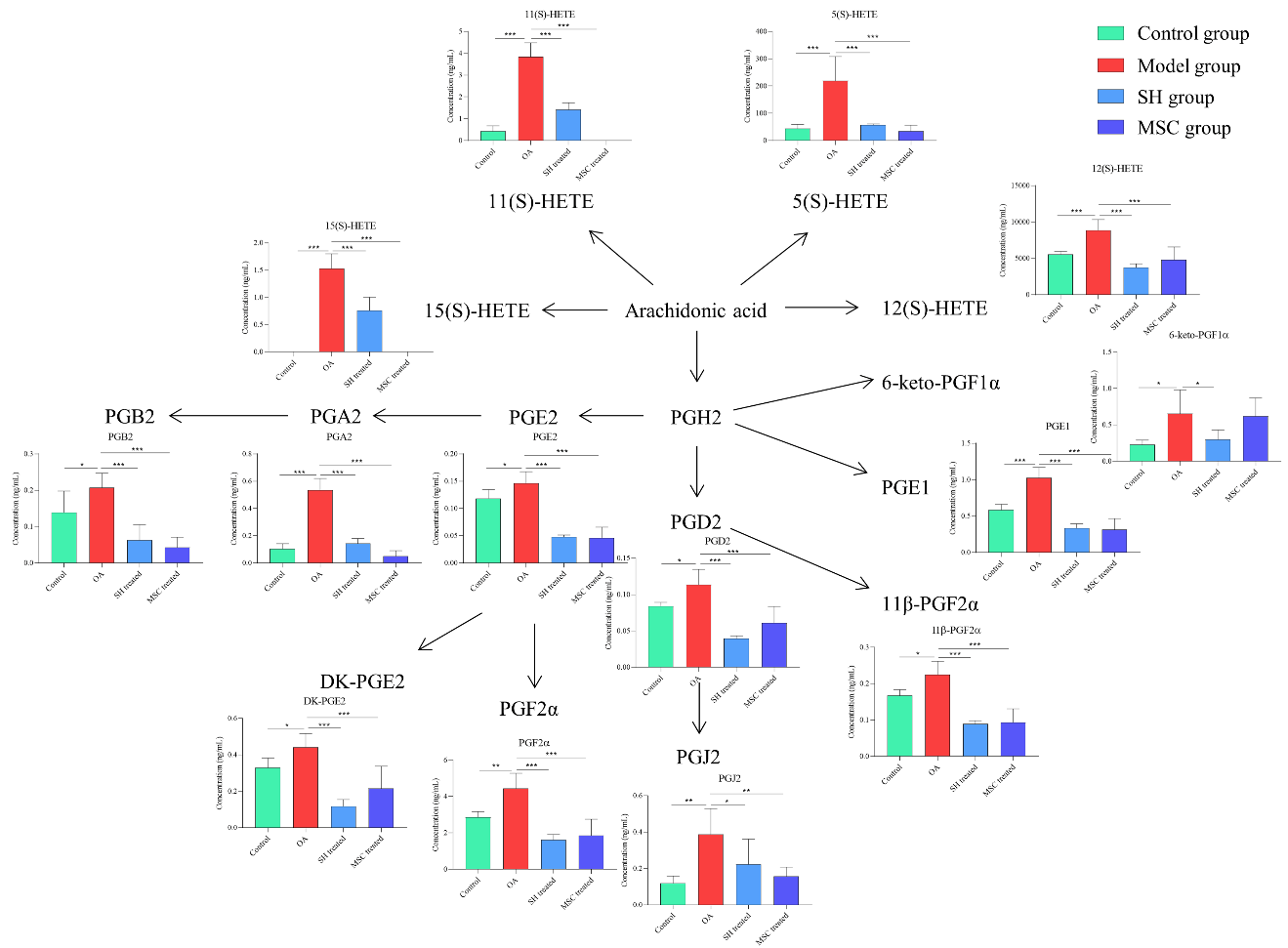


**Figure S2.** The metabolic pathway of PUFAs and their changes among different groups.

**Table S1**. The different concentrations (ng/mL) of PUFAs.

| **No.** | **Name** | **Concentrations (ng/mL)** | | | | | | | | | | | |
| --- | --- | --- | --- | --- | --- | --- | --- | --- | --- | --- | --- | --- | --- |
|  |  | **C1** | **C2** | **C3** | **C4** | **C5** | **C6** | **C7** | **C8** | **C9** | **C10** | **C11** | **C12** |
| 1 | 15(S)-HETE | 30 | 20.25 | 13.5 | 9 | 4.05 | 2.025 | 0.81 | 0.405 | 0.0405 | 0.0135 | 0.00405 | 0.00135 |
| 2 | 11(S)-HETE | 30 | 20.25 | 13.5 | 9 | 4.05 | 2.025 | 0.81 | 0.405 | 0.0405 | 0.0135 | 0.00405 | 0.00135 |
| 3 | 12(S)-HETE | 30 | 20.25 | 13.5 | 9 | 4.05 | 2.025 | 0.81 | 0.405 | 0.0405 | 0.0135 | 0.00405 | 0.00135 |
| 3 | 12(S)-HETE | 20000 | 10000 | 5000 | 2500 | 1250 | 500 | 250 | 125 | 50 | - | - | - |
| 4 | 5(S)-HETE | 30 | 20.25 | 13.5 | 9 | 4.05 | 2.025 | 0.81 | 0.405 | 0.0405 | 0.0135 | 0.00405 | 0.00135 |
| 4 | 5(S)-HETE | 800 | 400 | 200 | 100 | 50 | 25 | 10 | 5 | 2.5 | - | - | - |
| 5 | 15(S)-HEPE | 30 | 20.25 | 13.5 | 9 | 4.05 | 2.025 | 0.81 | 0.405 | 0.0405 | 0.0135 | 0.00405 | 0.00135 |
| 6 | 12(S)-HEPE | 30 | 20.25 | 13.5 | 9 | 4.05 | 2.025 | 0.81 | 0.405 | 0.0405 | 0.0135 | 0.00405 | 0.00135 |
| 7 | 5(S)-HEPE | 30 | 20.25 | 13.5 | 9 | 4.05 | 2.025 | 0.81 | 0.405 | 0.0405 | 0.0135 | 0.00405 | 0.00135 |
| 8 | PGA2 | 48 | 32.4 | 21.6 | 14.4 | 6.48 | 3.24 | 1.296 | 0.648 | 0.0648 | 0.0216 | 0.00648 | 0.00216 |
| 9 | PGB2 | 48 | 32.4 | 21.6 | 14.4 | 6.48 | 3.24 | 1.296 | 0.648 | 0.0648 | 0.0216 | 0.00648 | 0.00216 |
| 10 | PGJ2 | 48 | 32.4 | 21.6 | 14.4 | 6.48 | 3.24 | 1.296 | 0.648 | 0.0648 | 0.0216 | 0.00648 | 0.00216 |
| 11 | PGD2 | 60 | 40.5 | 27 | 18 | 8.1 | 4.05 | 1.62 | 0.81 | 0.081 | 0.027 | 0.0081 | 0.0027 |
| 12 | PGE2 | 108 | 72.9 | 48.6 | 32.4 | 14.58 | 7.29 | 2.916 | 1.458 | 0.1458 | 0.0486 | 0.01458 | 0.00486 |
| 13 | PGE1 | 60 | 40.5 | 27 | 18 | 8.1 | 4.05 | 1.62 | 0.81 | 0.081 | 0.027 | 0.0081 | 0.0027 |
| 14 | PGF2α | 60 | 40.5 | 27 | 18 | 8.1 | 4.05 | 1.62 | 0.81 | 0.081 | 0.027 | 0.0081 | 0.0027 |
| 15 | PGF2β | 60 | 40.5 | 27 | 18 | 8.1 | 4.05 | 1.62 | 0.81 | 0.081 | 0.027 | 0.0081 | 0.0027 |
| 16 | 6-keto-PGF1α | 400 | 270 | 180 | 120 | 54 | 27 | 10.8 | 5.4 | 0.54 | 0.18 | 0.054 | 0.018 |
| 17 | 11β-PGF2α | 60 | 40.5 | 27 | 18 | 8.1 | 4.05 | 1.62 | 0.81 | 0.081 | 0.027 | 0.0081 | 0.0027 |
| 18 | DK-PGE2 | 60 | 40.5 | 27 | 18 | 8.1 | 4.05 | 1.62 | 0.81 | 0.081 | 0.027 | 0.0081 | 0.0027 |
| 19 | 15(S)-HETrE | 30 | 20.25 | 13.5 | 9 | 4.05 | 2.025 | 0.81 | 0.405 | 0.0405 | 0.0135 | 0.00405 | 0.00135 |
| 20 | 12(S)-HHTrE | 400 | 270 | 180 | 120 | 54 | 27 | 10.8 | 5.4 | 0.54 | 0.18 | 0.054 | 0.018 |
| 20 | 12(S)-HHTrE | 8000 | 4000 | 2000 | 1000 | 500 | 250 | 125 | 50 | 25 | - | - | - |
| 21 | 13(S)-HOTrE | 30 | 20.25 | 13.5 | 9 | 4.05 | 2.025 | 0.81 | 0.405 | 0.0405 | 0.0135 | 0.00405 | 0.00135 |
| 22 | TXB2 | 400 | 270 | 180 | 120 | 54 | 27 | 10.8 | 5.4 | 0.54 | 0.18 | 0.054 | 0.018 |

**Table S2**. The gradient elution program of derivatized PUFAs using UHPLC-QQQ-MS analysis.

| **Time (min)** | **Solvent A (%)** | **Solvent B (%)** | **Flow (mL/min)** |
| --- | --- | --- | --- |
| 0 | 80 | 20 | 0.3 |
| 0.5 | 80 | 20 | 0.3 |
| 3 | 67 | 33 | 0.2 |
| 8 | 64 | 36 | 0.3 |
| 9 | 60 | 40 | 0.4 |
| 12.5 | 50 | 50 | 0.4 |
| 14 | 40 | 60 | 0.2 |
| 16 | 30 | 70 | 0.2 |
| 17 | 5 | 95 | 0.4 |
| 19.9 | 5 | 95 | 0.4 |
| 20 | 80 | 20 | 0.4 |

**Table S3.** The differential metabolites that had same changing trends by comparison of SH/mode groups with control/model group.

|  | **No.** | **Name** | **FC*** **(Ctrl/M)** | **p value (Ctrl/M)** | **FC***  **(SH/M)** | **p value (SH/M)** |
| --- | --- | --- | --- | --- | --- | --- |
| Arachidonic acid metabolism | 1 | Arachidonic acid | -1.043 | 0.51 | -1.15 | 0.0023 |
|  | 2 | 5(S)-HETE | -3.29 | 0.0084 | -3.15 | 9.67E-05 |
|  | 3 | 11(S)-HETE | -4.28 | 0.00019 | -2.20 | 4.60E-06 |
|  | 4 | 12(S)-HETE | -1.25 | 0.014 | -1.82 | 5.84E-08 |
|  | 5 | 15(S)-HETE | 0 | - | -1.75 | 0.0059 |
|  | 6 | PGA2 | -2.92 | 0.00017 | -2.81 | 1.75E-07 |
|  | 7 | PGB2 | -1.18 | 0.31 | -2.07 | 0.0011 |
|  | 8 | PGJ2 | -1.87 | 0.0062 | -1.41 | 0.010 |
|  | 9 | PGE2 | -1.18 | 0.085 | -2.63 | 1.63E-09 |
|  | 10 | PGD2 | -1.14 | 0.26 | -2.61 | 6.61E-08 |
|  | 11 | DK-PGE2 | -1.27 | 0.10 | -2.85 | 4.24E-07 |
|  | 12 | DK-PGD2 | -1.55 | 0.032 | -1.81 | 0.00033 |
|  | 13 | PGE1 | -1.03 | 0.83 | -2.38 | 5.25E-08 |
|  | 14 | PGF2β | -1.23 | 0.14 | -3.27 | 0.00028 |
|  | 15 | 11β-PGF2α | -1.30 | 0.040 | -1.99 | 1.28E-06 |
|  | 16 | PGF2α | -1.15 | 0.11 | -2.23 | 6.62E-08 |
|  | 17 | 6-keto-PGF1α | -1.98 | 0.11 | -1.74 | 0.0031 |
|  | 18 | TXB2 | -1.13 | 0.35 | -3.18 | 9.25E-08 |
|  | 19 | 5(S)-HEPE | 1.11 | 0.45 | 1.39 | 0.032 |
|  | 20 | 15(S)-HEPE | 2.00 | 0.0014 | 1.31 | 0.082 |
| α-linolenic acid and linoleic acid metabolism | 21 | Linolenic acid | -1.23 | 0.022 | -1.07 | 0.039 |
|  | 22 | Dihomo-γ-linolenic acid | -1.84 | 0.00012 | -1.68 | 2.97E-07 |
|  | 23 | Eicosapentaenoic acid | 1.74 | 0.00051 | 1.26 | 0.0041 |
|  | 24 | Docosahexaenoic acid | 1.27 | 0.00039 | 1.05 | 0.035 |
|  | 25 | Palmitoleate | -1.16 | 0.12 | -1.17 | 0.0013 |
|  | 26 | Docosadienoic acid | -4.69 | 9.45E-05 | -6.43 | 7.37E-09 |
|  | 27 | Docosatetraenoic acid | -1.21 | 0.073 | -1.68 | 1.18E-08 |
| Pyruvate metabolism | 28 | Malic acid | 1.37 | 0.34 | 1.95 | 0.031 |
| Bile acid biosynthesis | 29 | Cholic acid | -2.34 | 0.00050 | -3.61 | 7.86E-09 |
|  | 30 | Glycocholic acid | -1.53 | 0.010 | -2.80 | 1.45E-08 |
| Fatty acid biosynthesis | 31 | Acetic acid | 1.41 | 0.0011 | 1.25 | 0.0019 |
|  | 32 | Isobutyric acid | 1.02 | 0.82 | 1.36 | 0.012 |
|  | 33 | Myristic acid | -1.05 | 0.34 | -1.13 | 0.012 |
| Others | 34 | 235.0896 | 1.56 | 0.019 | 1.93 | 0.0029 |
|  | 35 | 271.2381 | -2.63 | 0.031 | -2.72 | 0.00082 |
|  | 36 | 285.3017 | 1.10 | 0.018 | 1.07 | 0.013 |
|  | 37 | 327.3376 | 1.23 | 0.035 | 1.34 | 0.013 |
|  | 38 | 372.3214 | -3.15 | 0.0098 | -1.66 | 0.010 |
|  | 39 | 410.2136 | -5.41 | 0.0092 | -3.70 | 0.00034 |
|  | 40 | 415.3017 | 1.50 | 0.23 | 3.98 | 0.0063 |
|  | 41 | 437.4466 | -1.15 | 0.14 | -1.27 | 9.83E-05 |
|  | 42 | 441.4408 | -2.07 | 2.50E-05 | -1.35 | 0.00041 |

* Fold change was evaluated by comparing sodium hyaluronate (SH) group with osteoarthritis guinea pigs (model) group, and healthy (control) group with model group. Fold change with a positive value indicates a relatively higher concentration present in SH group or control group, while a negative value means a relatively lower concentration as compared to the control group.

**Table S4.** The differential metabolites that had same changing trends by comparison of MSC/mode group with control/model group.

|  | **No.** | **Name** | **FC*** **(Ctrl/M)** | **p value (Ctrl/M)** | **FC*** **(MSC/M)** | **p value (MSC/M)** |
| --- | --- | --- | --- | --- | --- | --- |
| Arachidonic acid metabolism | 1 | 5(S)-HETE | -3.30 | 0.0085 | -5.10 | 1.87E-05 |
|  | 2 | 11(S)-HETE | -4.29 | 0.00019 | 0 | - |
|  | 3 | 12(S)-HETE | -1.25 | 0.014 | -1.38 | 0.0032 |
|  | 4 | 15(S)-HETE | 0 | - | 0 | - |
|  | 5 | PGA2 | -2.93 | 0.000174 | -8.93 | 2.92E-10 |
|  | 6 | PGB2 | -1.19 | 0.31 | -3.87 | 1.17E-07 |
|  | 7 | PGJ2 | -1.87 | 0.0062 | -1.85 | 7.39E-05 |
|  | 8 | PGE2 | -1.18 | 0.085 | -2.63 | 1.93E-08 |
|  | 9 | PGD2 | -1.15 | 0.26 | -1.62 | 0.00045 |
|  | 10 | DK-PGE2 | -1.28 | 0.10 | -1.50 | 0.015 |
|  | 11 | DK-PGD2 | -1.56 | 0.033 | -1.95 | 6.72E-05 |
|  | 12 | PGE1 | -1.03 | 0.83 | -2.38 | 4.64E-07 |
|  | 13 | PGF2β | -1.23 | 0.14 | -5.06 | 7.43E-07 |
|  | 14 | 11β-PGF2α | -1.31 | 0.040 | -1.79 | 5.27E-05 |
|  | 15 | PGF2α | -1.16 | 0.12 | -1.89 | 1.59E-05 |
|  | 16 | 6-keto-PGF1α | -1.98 | 0.11 | -1.48 | 0.037 |
|  | 17 | TXB2 | -1.13 | 0.35 | -3.17 | 2.47E-07 |
|  | 18 | 5(S)-HEPE | 1.11 | 0.51 | 1.32 | 0.043 |
|  | 19 | 15(S)-HEPE | 1.09 | 0.47 | 1.21 | 0.047 |
| α-linolenic and linoleic acid metabolism | 20 | Linolenic acid | -1.23 | 0.022 | -2.05 | 1.60E-12 |
|  | 21 | Dihomo-γ-linolenic acid | -1.85 | 0.00013 | -2.61 | 2.09E-10 |
|  | 22 | Eicosapentaenoic acid | 1.75 | 0.00052 | 1.23 | 0.035 |
|  | 23 | Docosahexaenoic acid | 1.27 | 0.00039 | 1.20 | 0.0040 |
|  | 24 | Palmitoleate | -1.15 | 0.11 | -1.68 | 4.55E-10 |
|  | 25 | Docosadienoic acid | -4.69 | 9.45E-05 | -5.07 | 1.88E-08 |
| Citric acid cycle | 26 | Malic acid | 1.37 | 0.35 | 4.01 | 2.07E-05 |
| Bile acid biosynthesis | 27 | Cholic acid | -2.34 | 0.00051 | -3.18 | 1.84E-08 |
|  | 28 | Glycocholic acid | -1.53 | 0.011 | -3.38 | 3.76E-09 |
| Others | 29 | 235.0896 | 1.56 | 0.020 | 1.69 | 0.023 |
|  | 30 | 271.2381 | -2.63 | 0.031 | -2.68 | 0.0019 |
|  | 31 | 410.2136 | -5.42 | 0.0093 | -10.87 | 6.04E-05 |
|  | 32 | 415.3017 | 1.50 | 0.23 | 2.23 | 0.00054 |
|  | 33 | 437.4466 | -1.16 | 0.14 | -1.84 | 5.24E-10 |
|  | 34 | 441.4408 | -2.08 | 2.51E-05 | -3.10 | 3.47E-11 |

* Fold change was evaluated by comparison of MSC group with model group, as well as control group with model group. Fold change with a positive value indicates a relatively higher concentration present in MSC group or control group, while a negative value means a relatively lower concentration as compared to the control group.

**Table S5**. The accuracy and precision of PUFAs using DIAAA derivatization-UHPLC-QQQ-MS.

| **Name** | **High concentration (C3 in Table S1)** | | | | **Middle concentration (C5 in Table S1)** | | | | **Low concentration (C7 in Table S1)** | | | |
| --- | --- | --- | --- | --- | --- | --- | --- | --- | --- | --- | --- | --- |
|  | **intraday** | | **interday** | | **intraday** | | **interday** | | **intraday** | | **interday** | |
|  | **Accuracy**  **(%)** | **Precision**  **(%)** | **Accuracy**  **(%)** | **Precision**  **(%)** | **Accuracy**  **(%)** | **Precision**  **(%)** | **Accuracy**  **(%)** | **Precision**  **(%)** | **Accuracy**  **(%)** | **Precision**  **(%)** | **Accuracy**  **(%)** | **Precision**  **(%)** |
| 15(S)-HETE | 99.00 | 2.18 | 103.27 | 7.31 | 96.53 | 0.72 | 91.87 | 2.62 | 84.43 | 4.68 | 83.37 | 5.97 |
| 11(S)-HETE | 96.30 | 0.80 | 99.60 | 6.92 | 92.50 | 1.57 | 94.73 | 5.95 | 82.83 | 4.16 | 83.13 | 7.72 |
| 12(S)-HETE | 99.87 | 3.43 | 102.33 | 8.06 | 96.30 | 0.88 | 92.77 | 7.64 | 87.07 | 3.27 | 86.90 | 8.39 |
| 5(S)-HETE | 102.97 | 1.49 | 103.80 | 6.42 | 100.90 | 3.80 | 100.47 | 2.18 | 91.07 | 2.83 | 90.20 | 7.92 |
| 15(S)-HEPE | 93.77 | 1.84 | 95.30 | 5.36 | 94.10 | 2.65 | 90.40 | 7.24 | 89.77 | 2.65 | 87.63 | 2.94 |
| 12(S)-HEPE | 94.93 | 0.30 | 98.60 | 7.11 | 97.13 | 1.31 | 96.63 | 5.88 | 91.40 | 2.96 | 89.50 | 6.57 |
| 5(S)-HEPE | 95.87 | 4.18 | 96.97 | 9.21 | 97.90 | 2.68 | 95.80 | 4.16 | 88.93 | 3.75 | 88.30 | 6.46 |
| PGA2 | 80.90 | 0.37 | 86.60 | 10.79 | 88.60 | 1.58 | 87.17 | 11.17 | 80.47 | 1.92 | 82.60 | 11.44 |
| PGB2 | 91.97 | 2.11 | 107.23 | 7.77 | 89.10 | 1.24 | 93.27 | 12.02 | 80.40 | 4.51 | 84.23 | 11.35 |
| PGJ2 | 106.00 | 2.04 | 116.60 | 8.62 | 107.00 | 2.96 | 115.47 | 10.03 | 97.07 | 0.95 | 104.87 | 10.34 |
| PGE2 | 99.23 | 1.23 | 98.50 | 1.65 | 88.87 | 2.26 | 87.43 | 0.79 | 96.07 | 1.03 | 94.33 | 3.13 |
| PGD2 | 93.40 | 5.23 | 87.40 | 13.25 | 91.00 | 0.78 | 81.53 | 14.80 | 89.80 | 2.32 | 78.90 | 10.96 |
| PGE1 | 106.57 | 5.31 | 106.60 | 4.49 | 97.07 | 2.13 | 95.23 | 3.98 | 87.20 | 1.07 | 86.57 | 3.23 |
| PGF2α | 105.80 | 2.26 | 111.00 | 7.65 | 97.60 | 1.59 | 100.93 | 7.09 | 91.37 | 2.85 | 89.97 | 6.40 |
| PGF2β | 106.00 | 1.29 | 112.20 | 6.59 | 92.97 | 4.61 | 91.50 | 5.37 | 90.67 | 1.06 | 90.80 | 4.25 |
| 6-keto PGF1α | 107.37 | 6.89 | 103.67 | 6.74 | 94.23 | 2.66 | 90.87 | 4.09 | 90.37 | 2.99 | 85.80 | 3.89 |
| 11β-PGF2α | 110.33 | 0.79 | 113.77 | 4.84 | 97.97 | 2.32 | 99.80 | 6.44 | 91.60 | 16.83 | 91.93 | 4.88 |
| 13,14-dihydro-15-  keto PGE2 | 101.07 | 1.85 | 97.87 | 8.97 | 96.13 | 3.85 | 93.63 | 2.72 | 91.63 | 1.48 | 84.37 | 1.44 |
| 15(S)-HETrE | 101.80 | 2.28 | 105.10 | 5.65 | 97.63 | 1.03 | 98.77 | 10.54 | 83.40 | 2.49 | 84.07 | 9.26 |
| 12(S)-HHTrE | 98.53 | 2.54 | 100.90 | 6.78 | 98.23 | 2.08 | 96.17 | 6.99 | 83.43 | 2.28 | 85.17 | 8.87 |
| 13(S)-HOTrE | 97.73 | 1.67 | 102.10 | 8.15 | 93.60 | 1.96 | 93.87 | 5.76 | 84.27 | 4.14 | 85.50 | 5.78 |
| TXB2 | 88.57 | 19.63 | 90.90 | 16.52 | 81.50 | 14.30 | 84.63 | 17.93 | 86.83 | 3.94 | 81.27 | 13.65 |

**Table S6**. Matrix effect and recovery of PUFAs using DIAAA derivatization-UHPLC-QQQ-MS.

| Name | High concentration | | Middle concentration | | Low concentration | |
| --- | --- | --- | --- | --- | --- | --- |
|  | Recovery (%) | Matrix effect (%) | Recovery (%) | Matrix effect (%) | Recovery (%) | Matrix effect (%) |
| 15(S)-HETE | 85.13 | 103.17 | 98.31 | 104.10 | 80.87 | 86.98 |
| 11(S)-HETE | 91.58 | 99.00 | 114.03 | 112.84 | 81.07 | 117.78 |
| 12(S)-HETE | 107.38 | 117.46 | 94.92 | 103.30 | 87.51 | 111.87 |
| 5(S)-HETE | 115.08 | 95.54 | 116.60 | 113.04 | 96.77 | 107.37 |
| 15(S)-HEPE | 86.60 | 98.29 | 111.55 | 107.90 | 92.49 | 111.95 |
| 12(S)-HEPE | 100.73 | 118.37 | 87.18 | 102.92 | 91.95 | 110.01 |
| 5(S)-HEPE | 90.46 | 114.27 | 91.86 | 100.57 | 86.19 | 92.78 |
| PGA2 | 81.03 | 85.91 | 81.21 | 86.78 | 87.68 | 82.91 |
| PGB2 | 82.29 | 81.05 | 86.59 | 81.46 | 103.02 | 100.70 |
| PGJ2 | 81.64 | 86.01 | 82.31 | 81.61 | 93.19 | 94.73 |
| PGD2 | 90.26 | 87.33 | 99.88 | 81.98 | 119.16 | 85.28 |
| PGE2 | 80.07 | 83.50 | 87.67 | 87.65 | 81.23 | 86.52 |
| PGE1 | 80.34 | 84.60 | 82.64 | 82.09 | 94.48 | 84.01 |
| PGF2α | 88.84 | 80.14 | 81.89 | 83.81 | 86.18 | 82.17 |
| PGF2β | 85.11 | 85.82 | 81.20 | 89.68 | 85.70 | 83.39 |
| 6-keto-PGF1α | 81.22 | 80.21 | 84.26 | 84.54 | 83.30 | 82.25 |
| 11β-PGF2α | 84.38 | 88.28 | 85.30 | 86.08 | 91.88 | 80.22 |
| DK-PGE2 | 82.58 | 85.46 | 86.76 | 81.11 | 80.15 | 109.18 |
| 15(S)-HETrE | 96.34 | 96.44 | 97.61 | 105.28 | 81.10 | 82.25 |
| 12(S)-HHTrE | 98.40 | 80.52 | 81.39 | 94.79 | 80.44 | 114.57 |
| 13(S)-HOTrE | 91.41 | 93.82 | 101.21 | 110.49 | 82.59 | 80.42 |
| TXB2 | 80.71 | 81.37 | 80.87 | 91.14 | 81.29 | 88.56 |

**Table S7**. The statistical analysis of PUFAs among control group and OA groups which were treated with SH, MSC or not.

| **Name** | **Fold change^*^ (Ctrl/M)** | **p value**  **(M *vs* Ctrl)** | **Fold change^*^ (SH/M)** | **p value**  **(M *vs* SH)** | **Fold change^*^ (MSC/M)** | **p value**  **(M *vs* MSC)** |
| --- | --- | --- | --- | --- | --- | --- |
| 15(S)-HETE |  |  | -2.01 | 3.48E-05 |  |  |
| 11(S)-HETE | -8.77 | 1.36E-06 | -2.69 | 1.34E-07 |  |  |
| 12(S)-HETE | -1.59 | 0.0013 | -2.35 | 1.70E-07 | -1.85 | 0.00027 |
| 5(S)-HETE | -4.96 | 0.0032 | -3.86 | 0.00013 | -6.24 | 0.00013 |
| 5(S)-HEPE | 1.12 | 0.42 | 1.70 | 0.0011 | 2.54 | 6.63E-06 |
| 12(S)-HEPE | 1.26 | 0.063 | 1.02 | 0.84 | 1.47 | 6.80E-05 |
| 15(S)-HEPE | 1.04 | 0.71 | 1.36 | 0.011 | 1.31 | 0.0016 |
| PGA2 | -5.09 | 2.31E-06 | -3.74 | 8.77E-09 | -10.60 | 3.48E-09 |
| PGB2 | -1.48 | 0.036 | -3.30 | 5.62E-06 | -4.84 | 4.67E-07 |
| PGJ2 | -3.25 | 0.0044 | -1.73 | 0.033 | -2.48 | 0.0013 |
| PGD2 | -1.35 | 0.021 | -2.88 | 1.04E-07 | -1.86 | 0.00042 |
| PGE2 | -1.23 | 0.042 | -3.06 | 2.85E-09 | -3.15 | 3.15E-07 |
| PGE1 | -1.75 | 0.00022 | -3.04 | 5.35E-09 | -3.24 | 4.01E-07 |
| PGF2α | -1.55 | 0.0052 | -2.74 | 3.7E-07 | -2.39 | 6.80E-05 |
| PGF2β | -1.13 | 0.32 | -3.88 | 7.19E-08 | -5.65 | 1.42E-07 |
| 6-keto-PGF1α | -2.87 | 0.028 | -2.20 | 0.011 | -1.06 | 0.81 |
| 11β-PGF2α | -1.34 | 0.014 | -2.51 | 6.96E-08 | -2.41 | 1.09E-05 |
| DK-PGE2 | -1.33 | 0.022 | -3.76 | 2.61E-08 | -2.05 | 0.00068 |
| 15(S)-HETrE | -1.11 | 0.26 | -1.54 | 0.00041 | -1.09 | 0.30 |
| 12(S)-HHTrE | -1.18 | 0.15 | -1.66 | 0.00032 | -1.17 | 0.24 |
| 13(S)-HOTrE | 1.11 | 0.33 | 2.09 | 2.88E-05 | 1.25 | 0.015 |
| TXB2 | -1.24 | 0.12 | -1.48 | 0.0032 | -1.59 | 0.0023 |

* Fold change was calculated from the mean values of each group. Fold change with a positive value indicates a relatively lower concentration present in model group compared with other groups, respectively, while a negative value means a relatively higher concentration present in model group compared with other groups, respectively.
